# First-line pembrolizumab with or without chemotherapy for recurrent or metastatic head and neck squamous cell carcinoma: 5-year follow-up of the Japanese population of KEYNOTE‑048

**DOI:** 10.1007/s10147-024-02632-x

**Published:** 2024-10-09

**Authors:** Nobuhiko Oridate, Shunji Takahashi, Kaoru Tanaka, Yasushi Shimizu, Yasushi Fujimoto, Koji Matsumoto, Tomoya Yokota, Tomoko Yamazaki, Masanobu Takahashi, Tsutomu Ueda, Nobuhiro Hanai, Hironori Yamaguchi, Hiroki Hara, Tomokazu Yoshizaki, Ryuji Yasumatsu, Masahiro Nakayama, Kiyoto Shiga, Takashi Fujii, Kenji Mitsugi, Kenichi Takahashi, Nijiro Nohata, Burak Gumuscu, Nati Lerman, Makoto Tahara

**Affiliations:** 1 Yokohama City University Graduate School of Medicine, 4-57 Urafune, Minami-ku, Yokohama, 236-0004 Japan; 2 Japanese Foundation for Cancer Research, 3-8-31, Ariake, Koto-ku, Tokyo 135-8500 Japan; 3 Kindai University Faculty of Medicine, 3-4-1 Kowakae, Higashiosaka City, Osaka 577-8502 Japan; 4 Hokkaido University Hospital, 5 Chome Kita 14 Jonishi, Kita Ward, Sapporo, Hokkaido 060-8648 Japan; 5 Aichi Medical University Hospital, Nagakute, Yazako, Karimata-1-1, Aichi, 480-1195 Japan; 6 Hyogo Cancer Center, 1370 Akashi, Hyogo 673-0021 Japan; 7 Shizuoka Cancer Center, 1007 Shimonagakubo, Nagaizumi, Sunto District, Shizuoka, 411-8777 Japan; 8 Miyagi Cancer Center, Nodayama-47-1 Medeshimashiote, Natori, Miyagi 981-1293 Japan; 9 Tohoku University Hospital, 1-1 Seiryomachi, Aoba Ward, Sendai, Miyagi 980-8574 Japan; 10 Hiroshima University Hospital, 1 Chome-2-3 Kasumi, Minami Ward, Hiroshima, 734-8551 Japan; 11 Aichi Cancer Center, 1-1 Kanokoden, Chikusa-ku, Nagoya 464-8681 Japan; 12 Jichi Medical University, 3311-1 Yakushiji, Shimotsuke, Tochigi 329-0498 Japan; 13 Saitama Cancer Center, 780 Komuro, Ina, Kitaadachi District, Saitama, 362-0806 Japan; 14 Kanazawa University, Kakumamachi, Kanazawa, Ishikawa 920-1192 Japan; 15 Kyushu University, 744 Motooka, Nishi-ku, Fukuoka, 819-0935 Japan; 16 University of Tsukuba, 1 Chome-1-1 Tennodai, Tsukuba, Ibaraki 305-8577 Japan; 17 Iwate Medical University, 19-1 Uchimaru, Morioka, Iwate 020-0023 Japan; 18 Osaka International Cancer Institute, 3-1-69 Otemae, Chuo-Ku, Osaka 541-8567 Japan; 19 Hamanomachi Hospital, 3-chōme-3-1 Nagahama, Chuo Ward, Fukuoka, 810-8539 Japan; 20 MSD K.K., Kitanomaru Square, 1-chōme-13-12 Kudankita, Chiyoda City, Tokyo 102-8667 Japan; 21 Merck & Co., Inc, 126 E. Lincoln Avenue, Rahway, NJ 07033 USA; 22 National Cancer Center Hospital East, 6-5-1 Kashiwanoha, Kashiwa, Chiba 277-8577 Japan

**Keywords:** Recurrent/metastatic head and neck squamous cell carcinoma, Pembrolizumab, PD-L1, Immunotherapy, EXTREME

## Abstract

**Background:**

Previously reported results from phase III KEYNOTE-048 demonstrated similar or improved overall survival (OS) with pembrolizumab or pembrolizumab-chemotherapy versus cetuximab-chemotherapy (EXTREME) in Japanese patients with recurrent/metastatic head and neck squamous cell carcinoma (R/M HNSCC). We report results in Japanese patients from KEYNOTE-048 after 5 years of follow-up.

**Methods:**

Patients with R/M HNSCC of the oropharynx, oral cavity, hypopharynx, or larynx were randomly assigned 1:1:1 to pembrolizumab, pembrolizumab-chemotherapy, or EXTREME. Primary endpoints were OS and progression-free survival. Efficacy was evaluated in the programmed cell death ligand 1 (PD-L1) combined positive score (CPS) ≥ 20, PD-L1 CPS ≥ 1, and total Japanese populations.

**Results:**

In Japan, 67 patients were enrolled (pembrolizumab, n = 23; pembrolizumab-chemotherapy, n = 25; EXTREME, n = 19). Median follow-up was 71.0 months (range, 61.2–81.5); data cutoff, February 21, 2022. 5-year OS rates with pembrolizumab versus EXTREME were 35.7% versus 12.5% (hazard ratio [HR] 0.38; 95% CI 0.13–1.05), 23.8% versus 12.5% (HR 0.70; 95% CI 0.34–1.45), and 30.4% versus 10.5% (HR 0.54; 95% CI 0.27–1.07) in the PD-L1 CPS ≥ 20, CPS ≥ 1, and total Japanese populations, respectively. 5-year OS rates with pembrolizumab-chemotherapy versus EXTREME were 20.0% versus 14.3% (HR 0.79; 95% CI 0.27–2.33), 10.5% versus 14.3% (HR 1.18; 95% CI 0.56–2.48), and 8.0% versus 12.5% (HR 1.11; 95% CI 0.57–2.16) in the PD-L1 CPS ≥ 20, CPS ≥ 1, and total Japanese populations, respectively.

**Conclusion:**

After 5 years of follow-up, pembrolizumab and pembrolizumab-chemotherapy showed long-term clinical benefits; results further support these treatments as first-line options for Japanese patients with R/M HNSCC.

**Clinical trial registration:**

NCT02358031.

**Supplementary Information:**

The online version contains supplementary material available at 10.1007/s10147-024-02632-x.

## Introduction

Head and neck squamous cell carcinoma (HNSCC) accounts for the majority of malignant tumors of the head and neck and are prevalent in Eastern Asia [1–4]. The epidermal growth factor receptor (EGFR) inhibitor cetuximab in combination with chemotherapy (platinum and 5-fluorouracil; EXTREME) was, until recently, the first-line standard of care for patients with recurrent or metastatic (R/M) HNSCC in many countries, including Japan [5–9]. The emergence of immune checkpoint inhibitors, however, has resulted in a paradigm shift in the management of patients with R/M HNSCC in both the first- and second-line treatment settings [10–12].

KEYNOTE-048 was a global phase III study evaluating pembrolizumab monotherapy and with chemotherapy (pembrolizumab-chemotherapy) versus EXTREME in patients with R/M HNSCC [12]. Pembrolizumab monotherapy significantly improved overall survival (OS) compared with EXTREME in patients with programmed cell death ligand 1 (PD-L1) combined positive score (CPS) ≥ 20 and CPS ≥ 1 and was associated with non-inferior OS in the total population. Pembrolizumab-chemotherapy significantly prolonged OS compared with EXTREME in all PD-L1 populations [12]. Based on these results, pembrolizumab was approved in Japan as a first-line treatment option as monotherapy or in combination with platinum and 5-fluorouracil for all patients with R/M HNSCC, regardless of PD-L1 CPS [13].

Given the high incidence of HNSCC in Japan [1, 3], it was important to investigate the efficacy and safety of pembrolizumab with or without chemotherapy in patients enrolled in KEYNOTE-048 from Japanese clinical centers [13]. With a follow-up of approximately 3 years, pembrolizumab improved median OS versus EXTREME in the PD-L1 CPS ≥ 20 population (28.2 versus 13.3 months; hazard ratio [HR], 0.29 [95% confidence interval (CI), 0.09–0.89]) and had similar OS in the PD-L1 CPS ≥ 1 and total Japanese populations [13]. Treatment with pembrolizumab-chemotherapy resulted in similar OS versus EXTREME in all populations.

Longer-term follow-up data from KEYNOTE-048 remain of interest to determine the durability of the OS benefit in patients with R/M HNSCC enrolled in Japan. Here, we report survival results after 5 years of follow-up in the Japanese population of KEYNOTE-048.

## Materials and methods

### Study design and participants

The design and methods of the open-label phase III KEYNOTE-048 study (ClinicalTrials.gov identifier NCT02358031) have been reported previously [12, 13]. Eligible patients were aged ≥ 18 years with previously untreated R/M squamous cell carcinoma of the oropharynx, oral cavity, hypopharynx, or larynx that was incurable by local therapy; had an Eastern Cooperative Oncology Group performance status (ECOG PS) of 0 or 1; had measurable disease per Response Evaluation Criteria in Solid Tumors version 1.1 (RECIST 1.1); and had known p16 expression for oropharyngeal cancers (non-oropharyngeal cancers were considered human papillomavirus [HPV] negative). Patients were randomly assigned 1:1:1 to pembrolizumab alone, pembrolizumab-chemotherapy, or EXTREME and stratified by percentage of tumor cells expressing PD-L1 (≥ 50% vs < 50%), HPV status for oropharyngeal cancers (p16 positive vs. p16 negative), and ECOG PS (0 vs 1).

The study protocol and amendments were approved by the appropriate institutional review boards or independent ethics committees at each center. The study was conducted in accordance with the protocol and Good Clinical Practice guidelines. All patients provided written informed consent.

### Procedures

In the pembrolizumab monotherapy and pembrolizumab-chemotherapy arms, pembrolizumab (200 mg) was administered once every 3 weeks. Chemotherapy in the pembrolizumab-chemotherapy and EXTREME arms comprised carboplatin (area under the curve 5 mg/mL/min) or cisplatin (100 mg/m^2^) and 5-fluorouracil (1000 mg/m^2^ per day for four consecutive days) every 3 weeks for six cycles. Patients in the EXTREME arm also received cetuximab (400-mg/m^2^ loading dose, then 250 mg/m^2^ per week). Pembrolizumab was administered until disease progression, intolerable toxicity, physician or participant decision, or completion of 35 cycles, whichever occurred first. After six cycles of EXTREME, patients could continue cetuximab monotherapy until progression, unacceptable toxicity, or withdrawal.

Imaging (computed tomography or magnetic resonance imaging) was performed at baseline, Week 9, and every 6 weeks until Year 1 and then every 9 weeks thereafter. Response was assessed per RECIST 1.1 by blinded independent central review. Survival was assessed every 12 weeks after confirmed disease progression or start of new anticancer therapy. Patients were monitored for adverse events (AEs) throughout treatment and for 30 days after stopping treatment (90 days for serious AEs). AEs were graded using the National Cancer Institute Common Terminology Criteria for Adverse Events version 4.0.

Baseline PD-L1 expression was assessed in archival or newly obtained tissue samples at a central laboratory using PD-L1 IHC 22C3 pharmDx (Agilent Technologies, Santa Clara, CA, USA). PD-L1 expression was characterized by CPS, defined as the number of PD-L1–staining cells (tumor cells, lymphocytes, and macrophages) divided by the total number of viable tumor cells multiplied by 100.

### Outcomes

Primary endpoints were OS and progression-free survival (PFS). OS was defined as the time from randomization to death from any cause. PFS was defined as the time from randomization to radiographically confirmed disease progression or death from any cause, whichever occurred first. Secondary endpoints included objective response rate (ORR), defined as the proportion of patients who have a complete response (CR) or partial response (PR), and safety. Duration of response (DOR), defined as the time from the first documented evidence of CR or PR until disease progression or death, was an exploratory endpoint. Efficacy was evaluated in patients with PD-L1 CPS ≥ 20, patients with PD-L1 CPS ≥ 1, and in the total Japanese population.

### Statistical analysis

OS, PFS, and ORR were assessed in all patients allocated to treatment (intention-to-treat population). DOR was assessed in all patients with a confirmed CR or PR. Safety was assessed in all patients who received ≥ 1 dose of study treatment. OS, PFS, and DOR were estimated using the Kaplan–Meier method. HRs and 95% CIs were calculated using a Cox proportional hazards regression model with Efron’s method of tie handling with treatment as a covariate. Statistical analyses were performed using SAS version 9.4 (SAS Institute Inc, Cary, NC, USA). No formal statistical hypothesis testing was performed for this subgroup analysis.

## Results

Of 882 patients enrolled in KEYNOTE-048, 67 were enrolled in Japan (pembrolizumab, n = 23; pembrolizumab-chemotherapy, n = 25; EXTREME, n = 19). Patient disposition, reasons for treatment discontinuation, and baseline characteristics (including differences between the treatment groups) have been reported previously [13]. The median follow-up (time from randomization to data cutoff [February 21, 2022]) was 71.0 months (range, 61.2–81.5).

After study drug discontinuation, subsequent anticancer therapy was received by 17 (73.9%), 11 (44.0%), and 14 (73.7%) patients in the pembrolizumab, pembrolizumab-chemotherapy, and EXTREME treatment groups, respectively (Online Resource 1). A subsequent EGFR inhibitor was received by 14 (60.9%), nine (36.0%), and two (10.5%) patients in the pembrolizumab, pembrolizumab-chemotherapy, and EXTREME treatment groups, respectively. Subsequent anti–PD-(L)1 therapy was received by two (8.7%), zero, and nine (47.4%) patients in the pembrolizumab, pembrolizumab-chemotherapy, and EXTREME treatment groups, respectively.

**Fig. 1:**
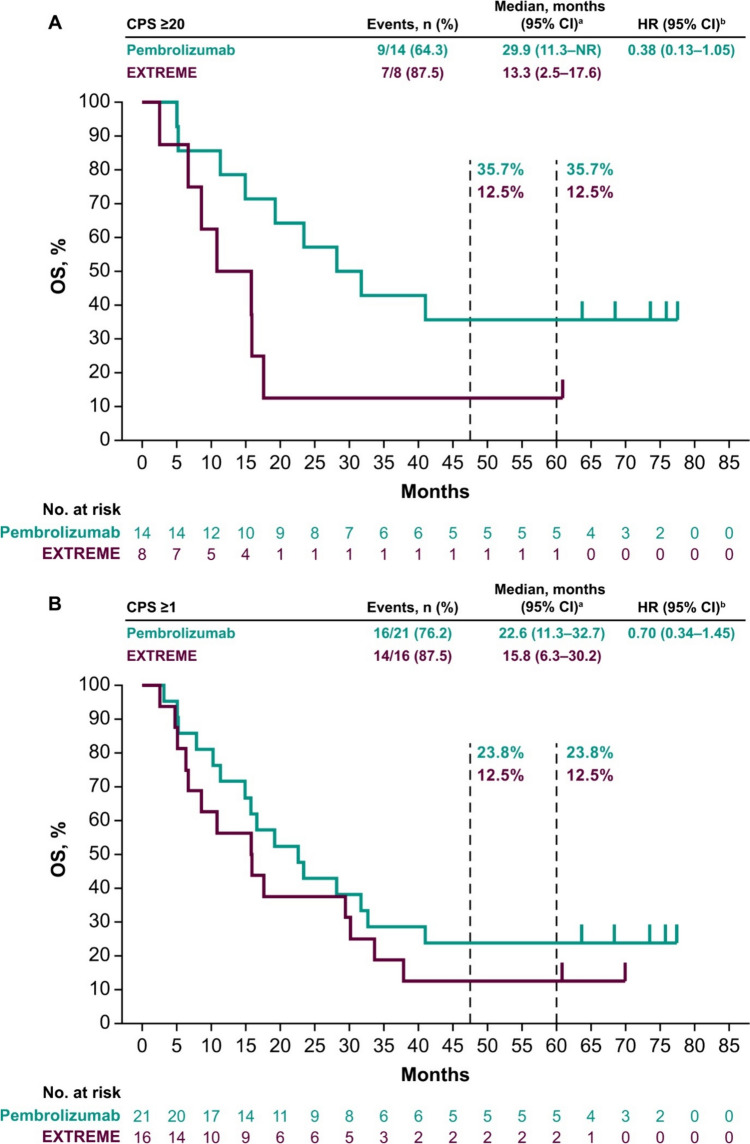

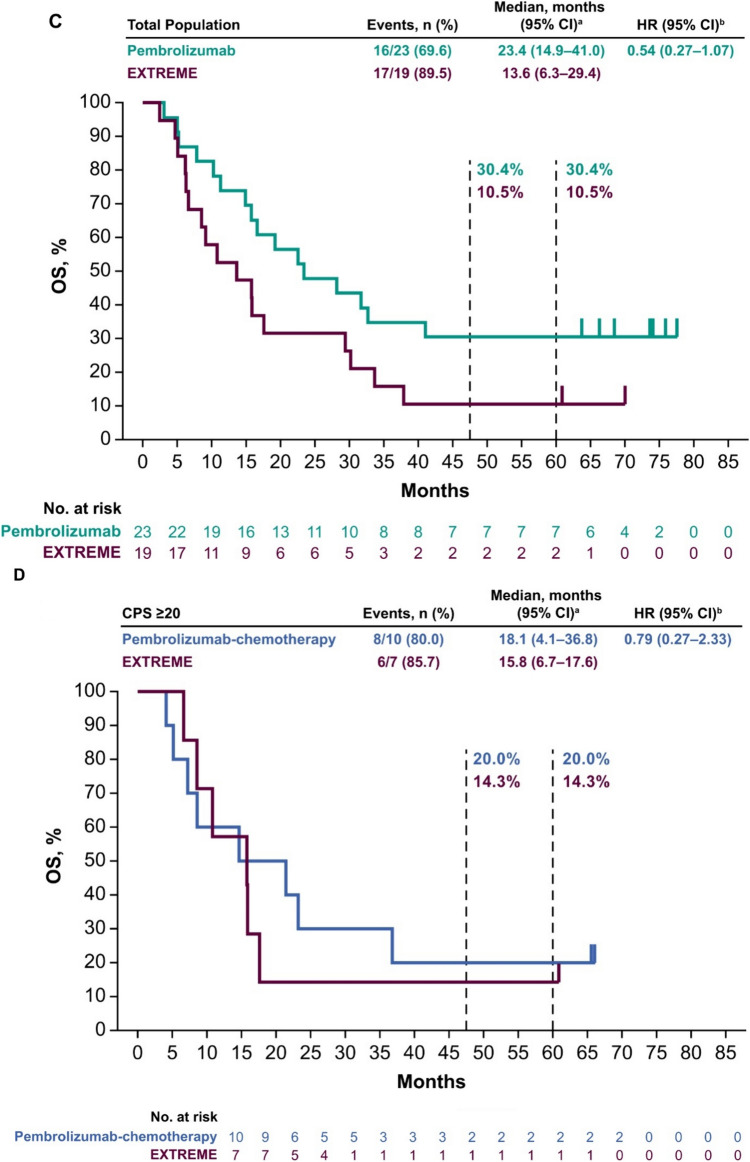

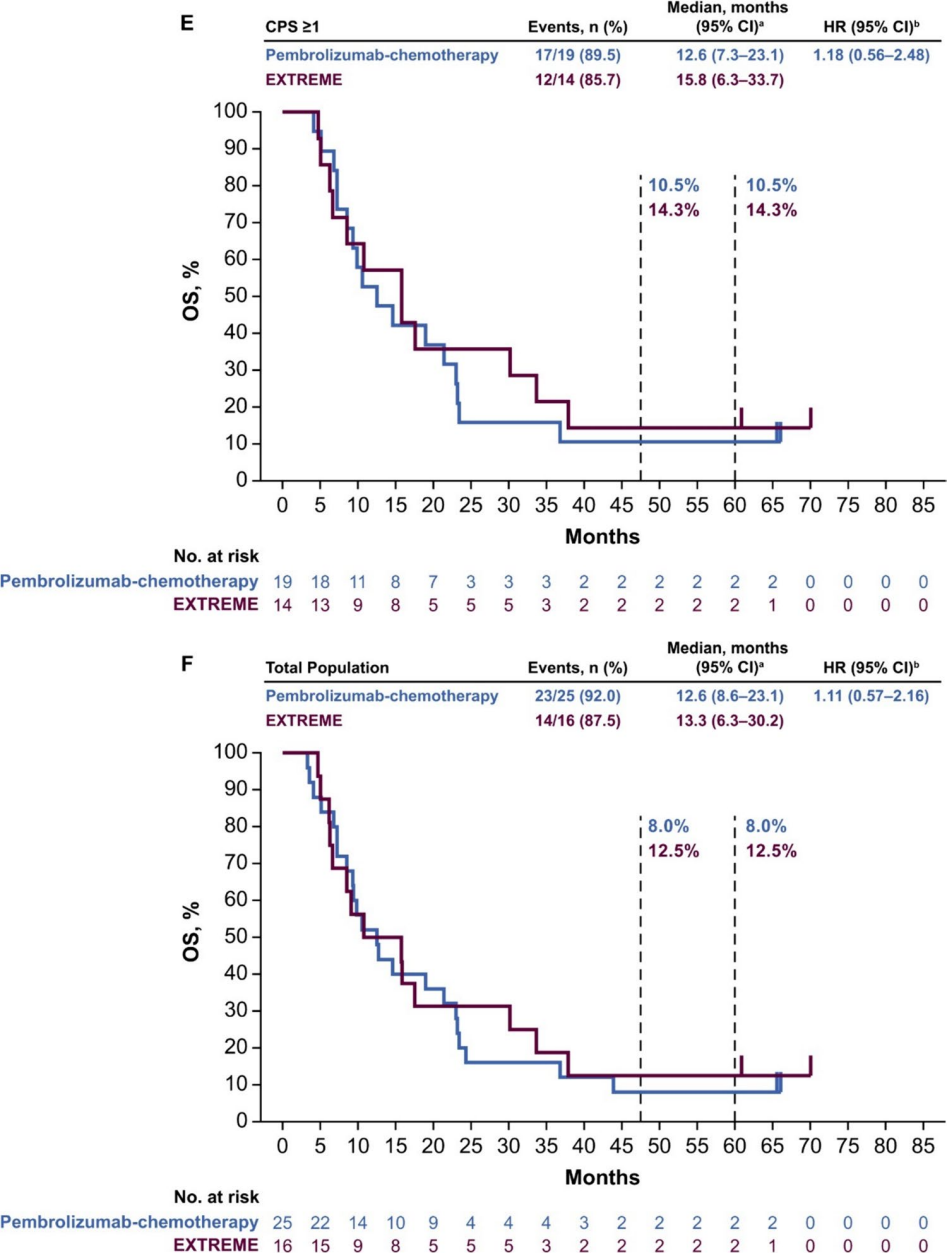
Pembrolizumab versus EXTREME in the **A** PD-L1 CPS ≥ 20, **B** PD-L1 CPS ≥ 1, and **C** total Japanese populations at long-term follow-up and pembrolizumab-chemotherapy versus EXTREME in the **D** PD-L1 CPS ≥ 20, **E** PD-L1 CPS ≥ 1, and **F** total Japanese populations. ^a^From the product-limit (Kaplan–Meier) method for censored data. ^b^On the basis of a Cox proportional hazards regression model with the Efron method of tie handling with treatment as a covariate. *CI* confidence interval, *CPS* combined positive score, *HR* hazard ratio, *OS* overall survival, *PD-L1* programmed cell death ligand 1

Median OS in the PD-L1 CPS ≥ 20 population was 29.9 months (95% CI 11.3 months–not reached) with pembrolizumab versus 13.3 months (95% CI 2.5–17.6 months) with EXTREME (HR 0.38; 95% CI 0.13–1.05) (Fig. 1A). In the PD-L1 CPS ≥ 1 population, median OS was 22.6 months (95% CI 11.3–32.7 months) with pembrolizumab versus 15.8 months (95% CI 6.3–30.2 months) with EXTREME (HR 0.70; 95% CI 0.34–1.45) (Fig. 1B). In the total Japanese population, median OS was 23.4 months (95% CI 14.9–41.0 months) with pembrolizumab versus 13.6 months (95% CI 6.3–29.4 months) with EXTREME (HR 0.54; 95% CI 0.27–1.07) (Fig. 1C). The 5-year OS rates with pembrolizumab versus EXTREME were 35.7% versus 12.5%, 23.8% versus 12.5%, and 30.4% versus 10.5% in the PD-L1 CPS ≥ 20, PD-L1 CPS ≥ 1, and total Japanese populations, respectively.

Median OS in the PD-L1 CPS ≥ 20 population was 18.1 months (95% CI 4.1–36.8 months) with pembrolizumab-chemotherapy versus 15.8 months (95% CI 6.7–17.6 months) with EXTREME (HR 0.79; 95% CI 0.27–2.33) (Fig. 1D). In the PD-L1 CPS ≥ 1 population, median OS was 12.6 months (95% CI 7.3–23.1 months) with pembrolizumab-chemotherapy versus 15.8 months (95% CI 6.3–33.7 months) with EXTREME (HR 1.18; 95% CI 0.56–2.48) (Fig. 1E). In the total Japanese population, median OS was 12.6 months (95% CI 8.6–23.1 months) with pembrolizumab-chemotherapy versus 13.3 months (95% CI 6.3–30.2 months) with EXTREME (HR 1.11; 95% CI 0.57–2.16) (Fig. 1F). The 5-year OS rates with pembrolizumab-chemotherapy versus EXTREME were 20.0% versus 14.3%, 10.5% versus 14.3%, and 8.0% versus 12.5% in the PD-L1 CPS ≥ 20, PD-L1 CPS ≥ 1, and total Japanese populations, respectively.

**Fig. 2:**
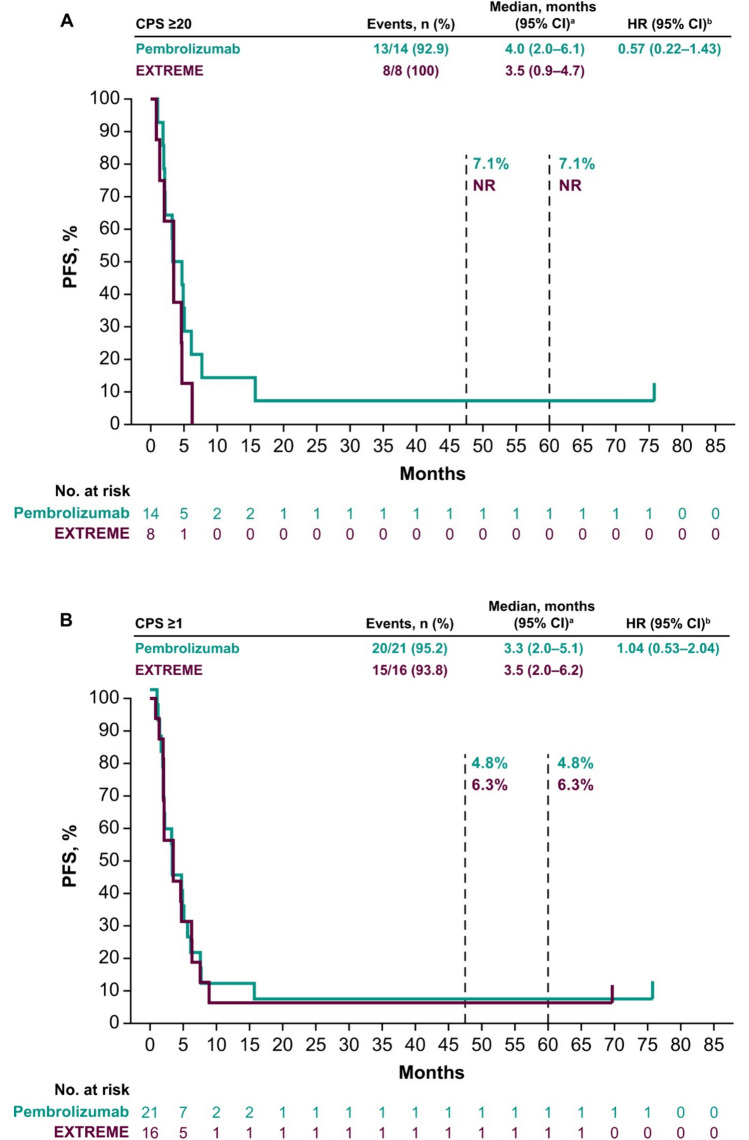

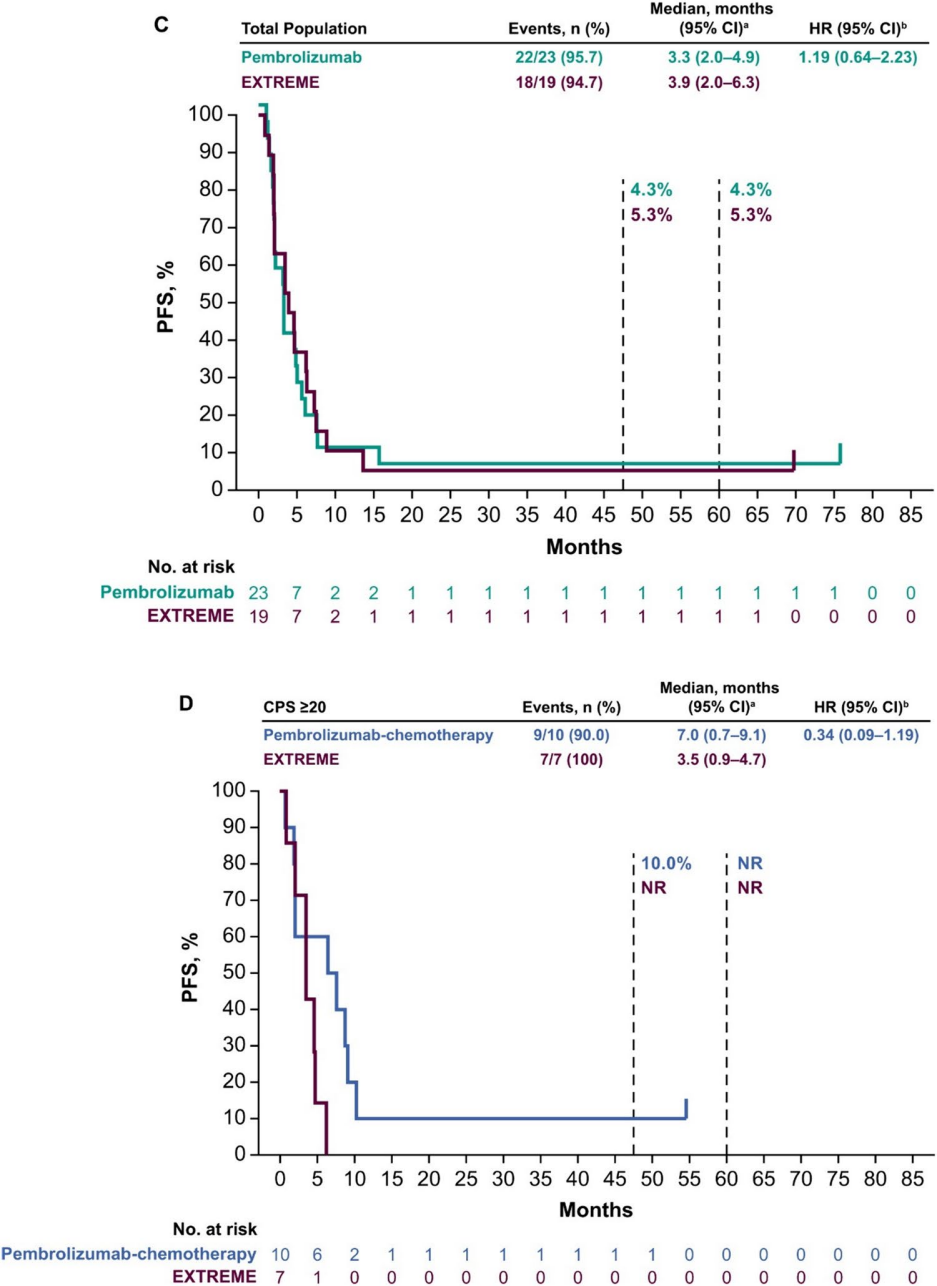

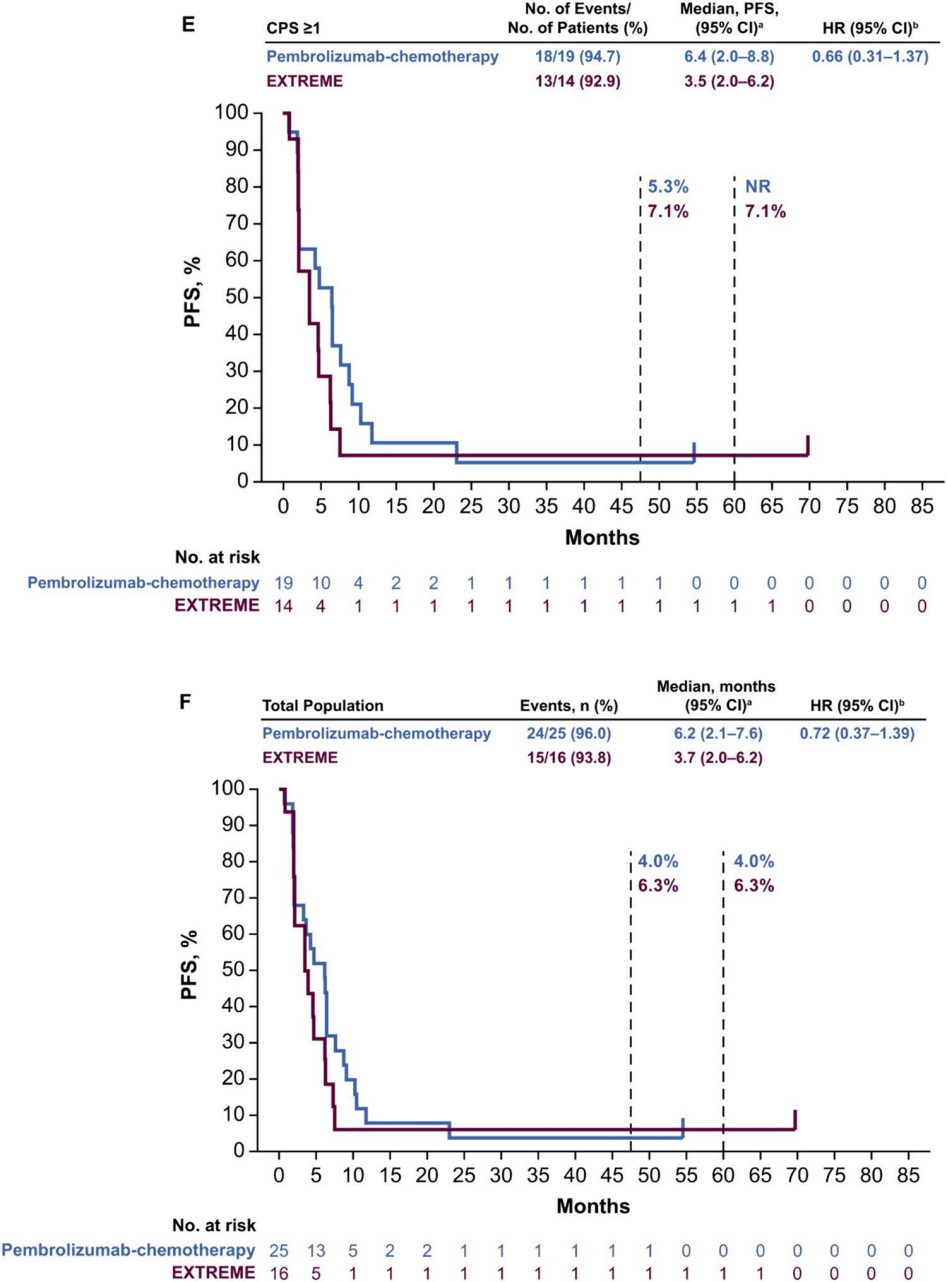
Pembrolizumab versus EXTREME in the **A** PD-L1 CPS ≥ 20, **B** PD-L1 CPS ≥ 1, and **C** total Japanese populations at long-term follow-up and pembrolizumab-chemotherapy versus EXTREME in the **D** PD-L1 CPS ≥ 20, **E** PD-L1 CPS ≥ 1, and **F** total Japanese populations. ^a^From the product-limit (Kaplan–Meier) method for censored data. ^b^On the basis of a Cox proportional hazards regression model with the Efron’s method of tie handling with treatment as a covariate. *CI* confidence interval, *CPS* combined positive score, *HR* hazard ratio, *PD-L1* programmed cell death ligand 1, *PFS* progression-free survival

Median PFS in the PD-L1 CPS ≥ 20 population was 4.0 months (95% CI 2.0–6.1 months) with pembrolizumab versus 3.5 months (95% CI 0.9–4.7 months) with EXTREME (HR 0.57; 95% CI 0.22–1.43) (Fig. 2A). In the PD-L1 CPS ≥ 1 population, median PFS was 3.3 months (95% CI 2.0–5.1 months) with pembrolizumab versus 3.5 months (95% CI 2.0–6.2 months) with EXTREME (HR 1.04; 95% CI 0.53–2.04 months) (Fig. 2B). In the total Japanese population, median PFS was 3.3 months (95% CI 2.0–4.9 months) with pembrolizumab versus 3.9 months (95% CI 2.0–6.3 months) with EXTREME (HR 1.19; 95% CI 0.64–2.23) (Fig. 2C).

Median PFS in the PD-L1 CPS ≥ 20 population was 7.0 months (95% CI 0.7–9.1 months) with pembrolizumab-chemotherapy versus 3.5 months (95% CI 0.9–4.7 months) with EXTREME (HR 0.34; 95% CI 0.09–1.19) (Fig. 2D). In the PD-L1 CPS ≥ 1 population, median PFS was 6.4 months (95% CI 2.0–8.8 months) with pembrolizumab-chemotherapy versus 3.5 months (95% CI 2.0–6.2 months) with EXTREME (HR 0.66; 95% CI 0.31–1.37) (Fig. 2E). In the total Japanese population, median PFS was 6.2 months (95% CI 2.1–7.6 months) with pembrolizumab-chemotherapy versus 3.7 months (95% CI 2.0–6.2 months) with EXTREME (HR 0.72; 95% CI 0.37–1.39) (Fig. 2F).

The ORR in the PD-L1 CPS ≥ 20 population was 28.6% (one CR, three PRs) with pembrolizumab versus 12.5% (one PR) with EXTREME, and median DOR was 8.4 versus 2.6 months (Table 1). In the PD-L1 CPS ≥ 1 population, the ORR was 19.0% (one CR, three PRs) with pembrolizumab versus 25.0% (one CR, three PRs) with EXTREME, and median DOR was 8.4 versus 5.5 months. In the total Japanese population, the ORR was 17.4% (one CR, three PRs) with pembrolizumab versus 36.8% (one CR, six PRs) with EXTREME, and median DOR was 8.4 versus 4.1 months.

**Table 1:** Summary of confirmed objective response and duration of response (per RECIST 1.1 by BICR) by PD-L1 CPS population with pembrolizumab versus EXTREME in the Japanese population

	PD-L1 CPS ≥ 20		PD-L1 CPS ≥ 1		Total population	
	Pembrolizumab (n = 14)	EXTREME (n = 8)	Pembrolizumab (n = 21)	EXTREME (n = 16)	Pembrolizumab (n = 23)	EXTREME (n = 19)
ORR	4 (28.6; 8.4–58.1)	1 (12.5; 0.3–52.7)	4 (19.0; 5.4–41.9)	4 (25.0; 7.3–52.4)	4 (17.4; 5.0–38.8)	7 (36.8; 16.3–61.6)
Best overall response						
CR	1 (7.1)	0	1 (4.8)	1 (6.3)	1 (4.3)	1 (5.3)
PR	3 (21.4)	1 (12.5)	3 (14.3)	3 (18.8)	3 (13.0)	6 (31.6)
SD	5 (35.7)	5 (62.5)	8 (38.1)	6 (37.5)	9 (39.1)	6 (31.6)
PD	5 (35.7)	2 (25.0)	9 (42.9)	6 (37.5)	10 (43.5)	6 (31.6)
Median (range) duration of response, months	8.4 (3.2–73.8 +)	2.6	8.4 (3.2–73.8 +)	5.5 (2.6–66.5 +)	8.4 (3.2–73.8 +)	4.1 (2.0–66.5 +)

Data are n (%; 95% CI) or n (%). ORR was defined as a best overall response of CR or PR. Duration of response was defined as the time from the first documented evidence of CR or PR until disease progression or death (whichever occurred first)

*BICR* blinded independent central review, *CI* confidence interval, *CPS* combined positive score, *CR* complete response; *ORR* objective response rate, *PD* progressive disease, *PD-L1* programmed cell death ligand 1, *PR* partial response, *RECIST* Response Evaluation Criteria in Solid Tumors, *SD* stable disease

^a^From product-limit (Kaplan–Meier) method for censored data; “ + ” indicates that there was no progressive disease by the time of last disease assessment

The ORR in the PD-L1 CPS ≥ 20 population was 50.0% (one CR, four PRs) with pembrolizumab-chemotherapy versus 14.3% (one PR) with EXTREME, and median DOR was 6.9 versus 2.6 months (Table 2). In the PD-L1 CPS ≥ 1 population, the ORR was 31.6% (one CR, five PRs) with pembrolizumab-chemotherapy versus 21.4% (one CR, two PRs) with EXTREME, and median DOR was 7.5 versus 4.1 months. In the total Japanese population, the ORR was 32.0% (one CR, seven PRs) with pembrolizumab-chemotherapy versus 31.3% (one CR, four PRs) with EXTREME, and median DOR was 7.5 versus 4.1 months.

**Table 2:** Summary of confirmed objective response and duration of response (per RECIST 1.1 by BICR) by PD-L1 CPS population with pembrolizumab-chemotherapy versus EXTREME in the Japanese population

	PD-L1 CPS ≥ 20		PD-L1 CPS ≥ 1		Total population	
	Pembrolizumab-chemotherapy (n = 10)	EXTREME (n = 7)	Pembrolizumab-chemotherapy (n = 19)	EXTREME (n = 14)	Pembrolizumab-chemotherapy (n = 25)	EXTREME (n = 16)
ORR	5 (50.0; 18.7–81.3)	1 (14.3; 0.4–57.9)	6 (31.6; 12.6–56.6)	3 (21.4; 4.7–50.8)	8 (32.0; 14.9–53.5)	5 (31.3; 11.0–58.7)
Best overall response						
CR	1 (10.0)	0	1 (5.3)	1 (7.1)	1 (4.0)	1 (6.3)
PR	4 (40.0)	1 (14.3)	5 (26.3)	2 (14.3)	7 (28.0)	4 (25.0)
SD	1 (10.0)	5 (71.4)	5 (26.3)	6 (42.9)	7 (28.0)	6 (37.5)
PD	4 (40.0)	1 (14.3)	7 (36.8)	5 (35.7)	8 (32.0)	5 (31.3)
Non-CR/non-PD	0	0	0	0	1 (4.0)	0
Not evaluable	0	0	1 (5.3)	0	1 (4.0)	0
Median (range) duration of response, months	6.9 (5.7–52.5 +)	2.6 (2.6–2.6)	7.5 (5.7–52.5 +)	4.1 (2.6–66.5 +)	7.5 (4.1–52.5 +)	4.1 (2.0–66.5 +)

Data are n (%; 95% CI) or n (%). Objective response rate was defined as a best overall response of CR or PR. Duration of response was defined as the time from the first documented evidence of CR or PR until disease progression or death (whichever occurred first)

*BICR* blinded independent central review, *CI* confidence interval, *CPS* combined positive score, *CR* complete response, *ORR* objective response rate, *PD* progressive disease, *PD-L1* programmed cell death ligand 1, *PR* partial response, *RECIST* Response Evaluation Criteria in Solid Tumors, *SD* stable disease

^a^From product-limit (Kaplan–Meier) method for censored data; “ + ” indicates that there was no progressive disease by the time of last disease assessment

**Fig. 3:**
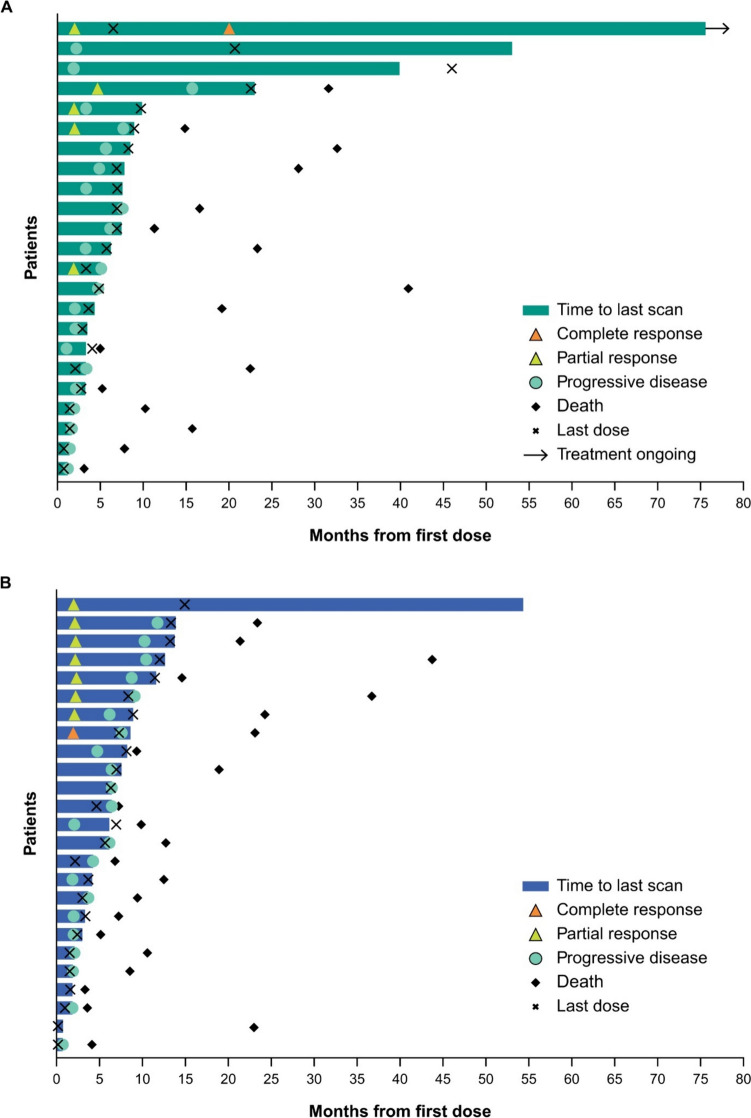

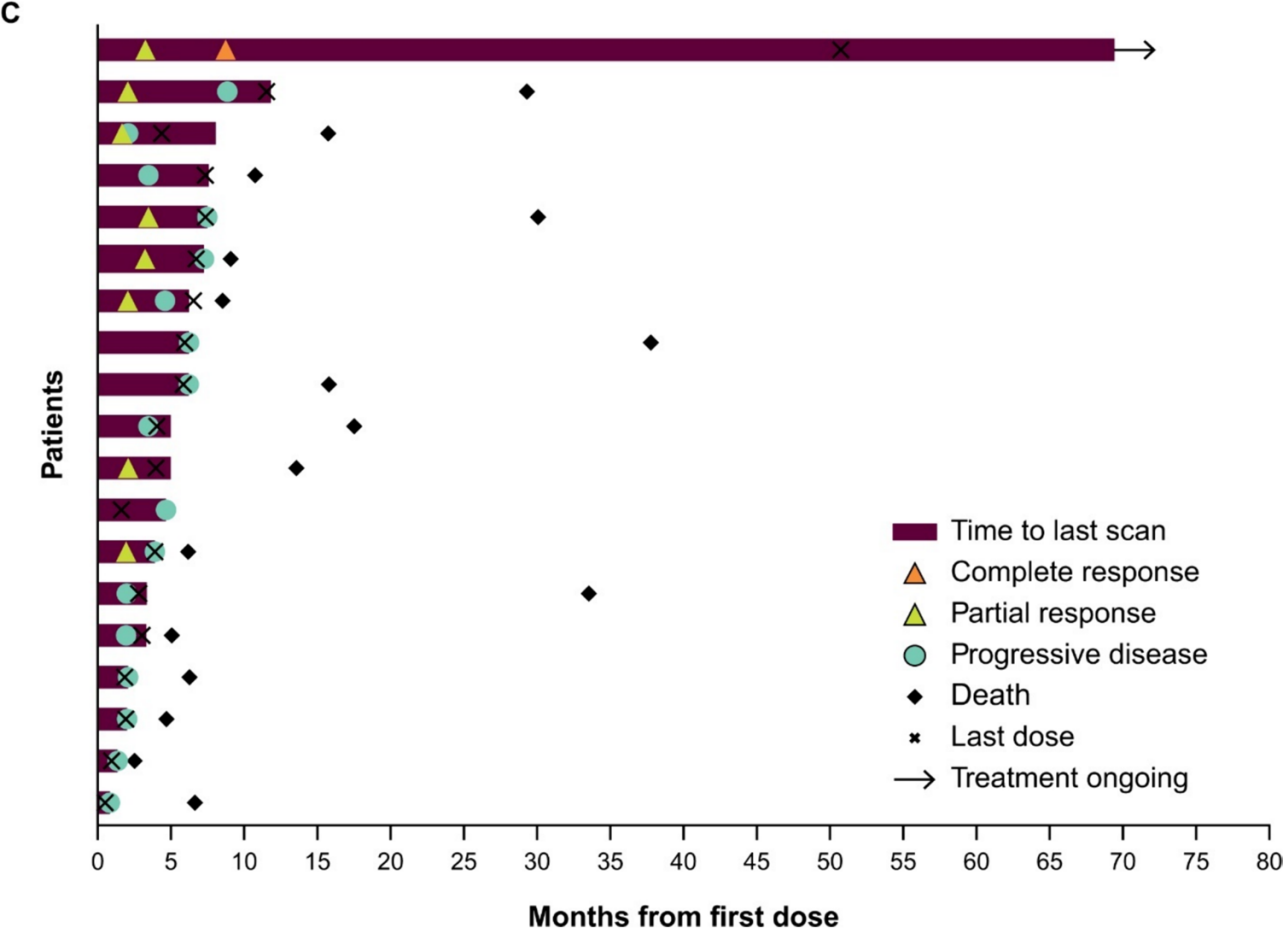
Time to response and response duration at long-term follow-up in the total Japanese population receiving **A** pembrolizumab, **B** pembrolizumab-chemotherapy, and **C** EXTREME

**Fig. 4:**
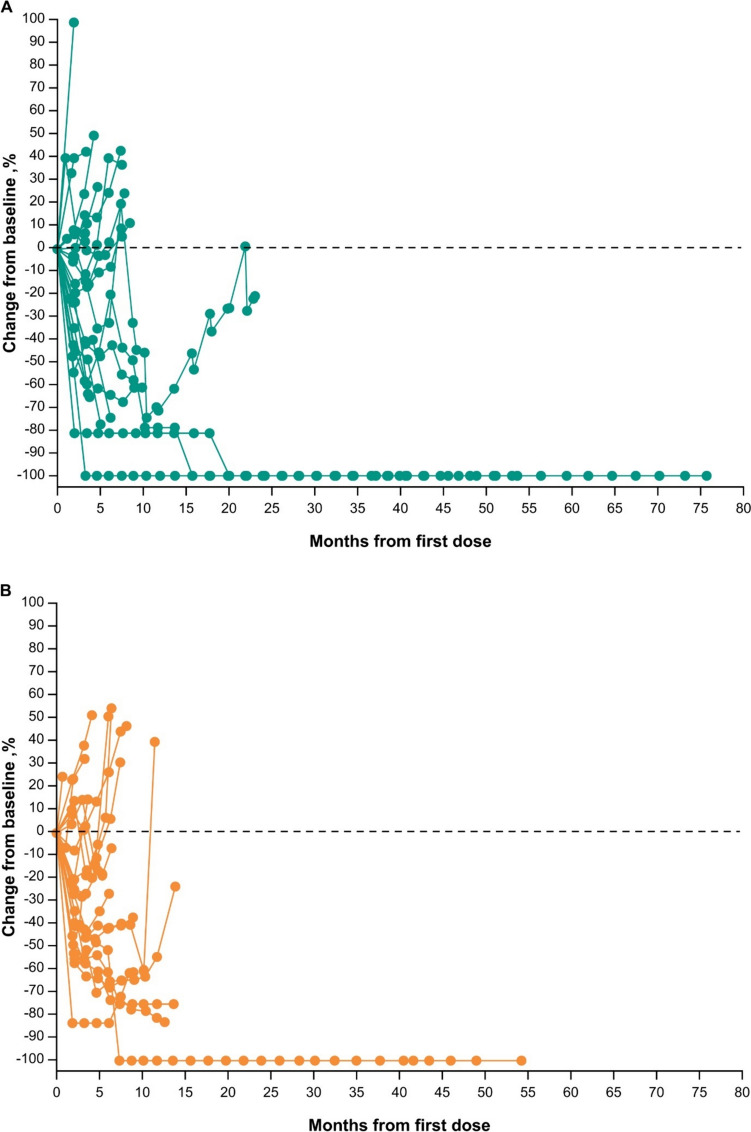

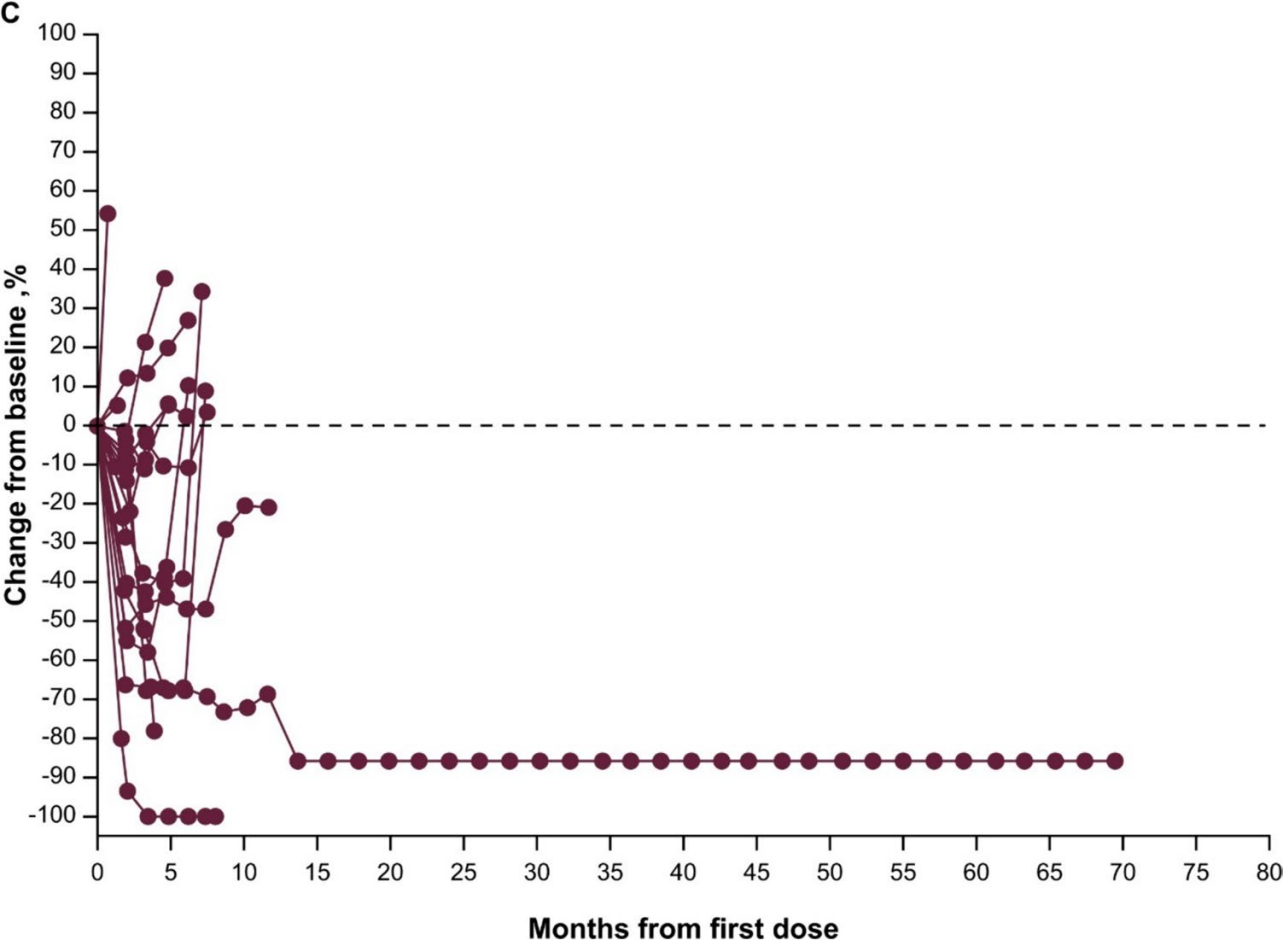
Change from baseline in target lesion size at long-term follow-up in the total Japanese population receiving **A** pembrolizumab monotherapy, **B** pembrolizumab-chemotherapy, and **C** EXTREME

Responses were durable with pembrolizumab (Fig. 3A), and response was ongoing in two patients with CR (one with pembrolizumab, one with EXTREME). Reductions in target lesion size from baseline were generally durable with all three treatments over time (Fig. 4A–C).

The median duration of treatment with pembrolizumab was 5.8 months (range, 0.7–24.0), and the median number of cycles administered was nine (range, 2–33). The median duration of treatment with pembrolizumab-chemotherapy was 5.7 months (range, 0.2–15.0), and the median number of cycles administered was seven (range, 1–22). The median duration of treatment with EXTREME was approximately 4.2 months (range, 0.5–51.0), and the median number of cycles administered was six (range, 1–52).

Detailed safety data for the Japanese population have been published previously [13]. Any-grade treatment-related AEs occurred in 17 (73.9%) patients in the pembrolizumab alone group, 25 (100%) in the pembrolizumab-chemotherapy group, and 19 (100%) in the EXTREME group (Table 3). Grade ≥ 3 treatment-related AEs were reported in five (21.7%), 19 (76.0%), and 17 (89.5%) patients, respectively. As reported previously, one Japanese patient receiving pembrolizumab-chemotherapy died because of treatment-related pneumonitis [13].

**Table 3:** Summary of treatment-related AEs in the Japanese population

	Pembrolizumab (n = 23)	Pembrolizumab-chemotherapy (n = 25)	EXTREME (n = 19)
Any treatment-related AEs^a^	17 (73.9)	25 (100)	19 (100)
Grade 3–5	5 (21.7)	19 (76.0)	17 (89.5)
Serious	3 (13.0)	7 (28.0)	5 (26.3)
Death	0	1 (4.0)	0
Discontinuation due to treatment-related AEs	2 (8.7)	1 (4.0)	4 (21.1)
Discontinuation due to serious treatment-related AEs	2 (8.7)	1 (4.0)	2 (10.5)

Data are n (%). Non-serious AEs within 30 days of the last dose and serious AEs within 90 days of the last dose are included. ^a^Determined by the investigator to be related to study treatment

*AE* adverse event

## Discussion

With an extended follow-up of 5 years, pembrolizumab monotherapy and pembrolizumab-chemotherapy conferred long-term clinical benefit as first-line treatment for Japanese patients with R/M HNSCC. While the number of patients enrolled in Japan was low and the populations were small, the results from the Japanese population are generally consistent with those from the global population in KEYNOTE-048 [14]. In the current analysis, pembrolizumab monotherapy improved OS compared with those treated with EXTREME in the total Japanese population. In Japanese patients with PD-L1 CPS ≥ 20, OS was numerically longer in patients treated with pembrolizumab-chemotherapy than in those treated with EXTREME. In patients in the PD-L1 CPS ≥ 1 population and in the total population, there was no apparent OS benefit in patients treated with pembrolizumab-chemotherapy compared with those treated with EXTREME. As reported previously [13], differences in baseline characteristics, such as ECOG PS, between treatment arms may have influenced the results of the current analysis, in addition to the higher number of patients in the EXTREME arm and the lower number of patients in the pembrolizumab-chemotherapy arm who received subsequent anti–PD-(L)1 therapy. Furthermore, the small sample size and wide confidence intervals limit interpretation of these results.

Survival rates observed in Japanese clinical practice are consistent with the 1-year rates we previously reported in the total Japanese population of KEYNOTE-048 [13] and in the global KEYNOTE-048 population [12]. The 1-year OS rates were 74% and 52% with pembrolizumab monotherapy and pembrolizumab-chemotherapy, respectively, in the total Japanese population [13] and 49% and 53%, respectively, in the total global population [12]. It is worth noting that real-world studies included patients with uncommon primary lesions, such as those in the paranasal sinuses and external auditory canal, who were excluded from KEYNOTE-048; outcomes in patients with HNSCC of the oropharynx, hypopharynx, oral cavity, and larynx in this real-world study were similar to those in KEYNOTE-048 [2, 15].

In conclusion, after 5 years of follow-up, first-line pembrolizumab monotherapy and pembrolizumab-chemotherapy showed long-term clinical benefit in Japanese patients with R/M HNSCC. Results from this study further support pembrolizumab and pembrolizumab-chemotherapy as first-line treatment options for Japanese patients with R/M HNSCC.

## Author contributors

Conceptualization: NN, BG. Data curation: YS, TYok, RY, NN, BG. Investigation: NO, ST, KT, KM, TYok, MT, NH, HY, HH, TYos, NM, TF, NN, BG, NL. Methodology: KT, NN, BG, NL. Project administration: ST, YF, KM, NH, HH. Formal analysis: KT, NN, BG. Writing—original draft preparation: NN, BG. Writing—review and editing: NO, ST, KT, YS, YF, KM, TYok, TYa, TU, NH, HH, NM, TF, KT, NN, BG, NL, MT. Funding acquisition: None. Resources: TU, KS, KM. Supervision: NO, BG, NL. Validation: KT, BG.

## Supplementary Information

Below is the link to the electronic supplementary material.

Supplementary file1 (DOCX 16 KB)

## Data Availability

Merck Sharp & Dohme LLC, a subsidiary of Merck & Co. Inc., Rahway, NJ, USA (MSD), is committed to providing qualified scientific researchers access to anonymized data and clinical study reports from the company’s clinical trials for the purpose of conducting legitimate scientific research. MSD is also obligated to protect the rights and privacy of trial participants and, as such, has a procedure in place for evaluating and fulfilling requests for sharing company clinical trial data with qualified external scientific researchers. The MSD data sharing website outlines the process and requirements for submitting a data request. Applications will be promptly assessed for completeness and policy compliance. Feasible requests will be reviewed by a committee of MSD subject matter experts to assess the scientific validity of the request and the qualifications of the requestors. In line with data privacy legislation, submitters of approved requests must enter into a standard data-sharing agreement with MSD before data access is granted. Data will be made available for request after product approval in the USA and elsewhere, or after product development is discontinued. There are circumstances that may prevent MSD from sharing requested data, including country or region-specific regulations. If the request is declined, it will be communicated to the investigator. Access to genetic or exploratory biomarker data requires a detailed, hypothesis-driven statistical analysis plan that is collaboratively developed by the requestor and MSD subject matter experts; after approval of the statistical analysis plan and execution of a data-sharing agreement, MSD will either perform the proposed analyses and share the results with the requestor or will construct biomarker covariates and add them to a file with clinical data that is uploaded to an analysis portal so that the requestor can perform the proposed analyses. For the MSD data sharing website, see https://engagezone.msd.com/ds_documentation.php.
