# Supplementary material for: First-line pembrolizumab with or without chemotherapy for recurrent or metastatic head and neck squamous cell carcinoma: 5-year follow-up of the Japanese population of KEYNOTE‑048
Source: Int J Clin Oncol. 2024 Oct 9;29(12):1825–39. doi: 10.1007/s10147-024-02632-x (PMC11588814; doi:10.1007/s10147-024-02632-x)
Supplement: Supplementary file 1 — Supplementary file1 (DOCX 16 KB) [file 10147_2024_2632_MOESM1_ESM.docx]

**Electronic Supplementary Material (online only)**

**Table of contents**

Online Resource 1 Subsequent anticancer therapies in the Japanese population.....2

**Online Resource 1** Subsequent anticancer therapies in the Japanese population^a^

|  | **Pembrolizumab  (n=23)** | **Pembrolizumab-chemotherapy  (n=25)** | **EXTREME  (n=19)** |
| --- | --- | --- | --- |
| Any | 17 (73.9) | 11 (44.0) | 14 (73.7) |
| Chemotherapy | 17 (73.9) | 10 (40.0) | 8 (42.1) |
| EGFR inhibitor | 14 (60.9) | 9 (36.0) | 2 (10.5) |
| Tyrosine kinase inhibitor | 0 | 2 (8.0) | 0 |
| Immune checkpoint inhibitor | 2 (8.7) | 0 | 9 (47.4) |
| Anti–PD-L1 or anti–PD-1 | 2 (8.7) | 0 | 9 (47.4) |
| Anti-CTLA4 | 0 | 0 | 0 |
| Anti–B7-H3 | 0 | 0 | 0 |
| Anti-TIGIT | 0 | 0 | 0 |

Data are n (%)

^a^Patients could have received ≥1 subsequent therapy

*B7-H3* B7 homolog 3 protein, *CTLA4* cytotoxic T-lymphocyte–associated protein 4, *EGFR* epidermal growth factor receptor, *PD-1* programmed cell death protein 1, *PD-L1* programmed cell death ligand 1, *TIGIT* T-cell immunoreceptor with immunoglobulin and ITIM domains
